# Degeneracy and Redundancy in Active Inference

**DOI:** 10.1093/cercor/bhaa148

**Published:** 2020-06-03

**Authors:** Noor Sajid, Thomas Parr, Thomas M Hope, Cathy J Price, Karl J Friston

**Affiliations:** Wellcome Centre for Human Neuroimaging, UCL Queen Square Institute of Neurology, London, WC1N 3AR, UK

**Keywords:** active inference, complexity, degeneracy, free energy, redundancy

## Abstract

The notions of degeneracy and redundancy are important constructs in many areas, ranging from genomics through to network science. Degeneracy finds a powerful role in neuroscience, explaining key aspects of distributed processing and structure–function relationships in the brain. For example, degeneracy accounts for the superadditive effect of lesions on functional deficits in terms of a “many-to-one” structure–function mapping. In this paper, we offer a principled account of degeneracy and redundancy, when function is operationalized in terms of active inference, namely, a formulation of perception and action as belief updating under generative models of the world. In brief, “degeneracy” is quantified by the “entropy” of posterior beliefs about the causes of sensations, while “redundancy” is the “complexity” cost incurred by forming those beliefs. From this perspective, degeneracy and redundancy are complementary: Active inference tries to minimize redundancy while maintaining degeneracy. This formulation is substantiated using statistical and mathematical notions of degenerate mappings and statistical efficiency. We then illustrate changes in degeneracy and redundancy during the learning of a word repetition task. Finally, we characterize the effects of lesions—to intrinsic and extrinsic connections—using in silico disconnections. These numerical analyses highlight the fundamental difference between degeneracy and redundancy—and how they score distinct imperatives for perceptual inference and structure learning that are relevant to synthetic and biological intelligence.

## Introduction

Degenerate functional architectures generally feature multiple pathways that are available to fulfill a particular functional endpoint (Tononi et al. 1999; Price and Friston 2002; Friston and Price 2003). A simple example would be that either the left or right hand could be used to “lift a cup.” This provides a degenerate structure–function relationship that preserves function following damage because, in this example, the ability to lift a cup is conserved if only one hand is damaged. The basic idea pursued in this paper is that degeneracy affords a flexibility that offsets the cost of redundancy. For example, being able to lift a cup with the right or left hand keeps “options open,” while using both hands would be redundant. Furthermore, when multiple functions can be supported by the same structures (i.e., when there is a many-to-many mapping between structure and function), the trade-off between degeneracy and redundancy becomes even more pronounced. For example, when conducting fine-control tasks like painting or surgery, is it more efficient for both hands to be equally dextrous or is one “preferred hand” sufficient? In what follows, we try to answer this question using notions of Bayes optimality inherent in active inference (Friston, FitzGerald, et al. 2017a).

Degeneracy is often mistakenly confused with redundancy (Whitacre and Bender 2010). Redundancy is the counterpart of “efficiency” (Barlow 1961, 1974; Laughlin 2001) and implies an inefficient use of a system’s degrees of freedom to achieve a particular functional endpoint. As noted above, it would be redundant to use both hands to “lift a cup,” when one was sufficient. The purpose of this paper is to disambiguate degeneracy and redundancy in formal and quantitative terms—and establish the validity of these definitions at complementary levels using the free energy principle. The free energy principle is, put simply, a technical way of articulating the Bayesian brain hypothesis (Knill and Pouget 2004; Daw et al. 2005; Doya et al. 2007; Friston 2009). In virtue of committing to the Bayesian brain hypothesis, we also commit to the complete class theorem (Wald 1947). This means that—by definition—any observable behavior under some specifiable loss function can be cast as free energy minimization.

In the first section, we consider the theoretical basis of degeneracy and redundancy in the context of the free energy principle and active inference (Friston et al. 2006). In short, degeneracy is formulated as the flexibility afforded by our internal explanations for sensory outcomes, while redundancy becomes the complexity cost incurred by constructing those explanations. By operationalizing function as such, we assume some functional constraints—on synthetic and biological intelligence supervenes. This section concludes by rehearsing some straightforward but revealing insights—under this formulation—about the nature of degeneracy and redundancy and how they stand in relation to each other.

The formulation presented provides a Bayesian formalism of the intuitions (Price and Friston 2002; Friston and Price 2003), natural (and principled) extensions to the mutual information (i.e., complexity) parameterization (Tononi et al. 1999; Man et al. 2016), and conceptual treatments of the fundamental rationale for degeneracy (i.e., self-organizing processes) in (Kelso 2012). It provides a potentially useful way of investigating degeneracy and redundancy—within synthetic and biological intelligence—beyond structural quantification.

The second section uses simulations and numerical analyses to illustrate how degeneracy and redundancy depend upon the structure of a generative model—and the implicit neuronal connectivity. As an illustrative example, we investigated the relationship between redundancy and degeneracy in a generative model of word repetition (i.e., the ability to repeat back a heard word). The key argument of this section rests on a series of model comparisons that quantify changes in degeneracy and redundancy following removal of particular connections or model parameters (i.e., in silico lesions). Specifically, we removed connections that were redundant in relation to the task at hand. This removal is consistent with formulations of redundancy in the context of synaptic homoeostasis (Tononi and Cirelli 2006) and the elimination of redundant model parameters during sleep (Hobson and Friston 2012). We hypothesized that removing redundant connections would increase model evidence by reducing redundancy, relative to degeneracy.

The third section focuses on another type of in silico lesion experiments: removal of nonredundant connections as might happen in pathological disorders. Here, we associate different sorts of pathology with a selective impairment of extrinsic (between-region) and intrinsic (within-region) connectivity. Our expectation was that lesions to both intrinsic and extrinsic connections would show a superadditive effect on behavioral performance. Here, superadditive refers to increased (negative) effects on accuracy as a result of a dual lesion, in contrast to combined effects of single lesions. Furthermore—due to tightly coupled connection between redundancy and degeneracy—we hoped to show that pathological disconnections “increased” redundancy—and that this increase was greater than the accompanying increases in degeneracy. Crucially, the effect of lesions can also be used to identify the neuronal correlates of belief updating. This allowed us to make some empirical predictions about electrophysiological responses to word repetition under different lesion loads.

We conclude with a brief discussion about the implications of this formulation of degeneracy (and redundancy) for active Bayesian inference in the brain and lesion studies.

## Degeneracy, Redundancy, and Active Inference

The definitions of degeneracy and redundancy on offer in this work rest upon a commitment to the free energy principle, namely, that the functional imperative for any sentient system—in particular the brain—is to minimize variational free energy (Friston 2019; Sajid et al. 2019). This is equivalent to maximizing the evidence for internal generative models of how sensations are caused (Dayan et al. 1995). This evidence is also known as the marginal likelihood. In short, brain function can be measured in terms of its ability to self-evidence (Hohwy 2016). There is a large literature on the free energy principle, active inference, and statistical physics that underwrites this formulation of action and perception in the brain, which is usually referred to as “active inference” (a.k.a. predictive processing) (Clark 2016). Here, variational free energy is an information theoretic construct that, while related to homologous concepts in physics, should not be interpreted directly in terms of metabolic energy.

For those people unfamiliar with the free energy principle, it can be read as a general principle that subsumes most (arguably all) normative theories of sentience and self-organization in the neurosciences, for example, the Bayesian brain hypothesis, predictive processing, active inference, reinforcement learning, Bayesian decision theory, universal computation, and treatments based upon information theory (i.e., the principle of minimum redundancy, the principle of maximum efficiency, and so on) (Barlow 1961; Optican and Richmond 1987; Sutton and Barto 1998; Knill and Pouget 2004; Hutter 2006; Doya et al. 2007; Gershman and Daw 2017; Stachenfeld et al. 2017). In other words, it is an optimality principle (based upon Hamilton’s principle of least action) that furnishes a description of perception and action in formal (information theoretic) terms. In and of itself, it is not central to the arguments of this paper—it simply provides a formal specification of “function” in the sense of “structure–function” relationships.

Central to this treatment is the notion of a “generative model” that can generate predictions of the sensory consequences of plausible causes—usually referred to as “hidden states” of the world. The form or structure of this generative model can then be adopted as the “structure” in understanding structure–function relationships. This structure underwrites functional brain architectures, which realize active inference (Friston and Buzsaki 2016). Our focus here is on how degeneracy and redundancy manifest in terms of active inference and implicit structure–function relationships. To understand the formal nature of degeneracy and redundancy, it is useful to unpack free energy in terms of its constituent parts. In what follows, we will use free energy and surprisal synonymously to refer to the negative logarithm of the probability of any sensory input under a generative model. This probability is the Bayesian model evidence or marginal likelihood that has to be maximized by any system that perceives, acts, and learns in a changing world (Friston 2019). Additionally, at a neuronal level, Isomura and Friston (2018) have shown that even in vitro neural networks minimize their (variational) free energy.

Statistically speaking, free energy can always be expressed in terms of “accuracy” and “complexity.” In other words, the log evidence associated with any pattern of sensory outcomes—at any point in time—can be separated into accuracy and complexity, where the (log) evidence is accuracy minus complexity. In terms of free energy:

$$ {\displaystyle \begin{array}{c}F={\mathbb{E}}_Q\left[\ln Q(s)-\ln P\left(s,o\right)\right]\\{}=\underset{\mathrm{evidence}\kern0.17em \mathrm{bound}}{\underbrace{D_{KL}\left[Q(s)\Big\Vert P\left(s|o\right)\right]}}-\underset{\log\ \mathrm{evidence}}{\underbrace{\ln P(o)}}\\{}=\underset{\mathrm{complexity}}{\underbrace{D_{KL}\left[Q(s)\Big\Vert P(s)\right]}}-\underset{\mathrm{accuracy}}{\underbrace{{\mathbb{E}}_Q\Big[\ln P\left(o|s\right)}}\Big]\\{}=\underset{\mathrm{energy}}{\underbrace{{\mathbb{E}}_Q\left[-\ln P\left(s,o\right)\right]}}-\underset{\mathrm{entropy}}{\underbrace{{\mathbb{E}}_Q\Big[-\ln Q(s)}}\Big]\end{array}} $$

These expressions show that free energy depends on a generative model *P*(*s*, *o*), which describes the relationships between (hidden) states (*s*) that cause (observed) outcomes (*o*), and posterior beliefs about the causes *Q*(*s*). This means that to self-evidence, it is necessary to find an accurate explanation for sensory observations that incurs the least complexity cost (as indicated by the third equality above). Formally, the accuracy is the expected log likelihood of the sensory outcomes, given some posterior beliefs about the causes of those data. Complexity is the difference between these posterior beliefs and prior beliefs, that is, prior to seeing the outcomes. In essence, complexity scores the degree to which posterior beliefs have to move away from prior beliefs to explain the data at hand. Alternatively, they can be thought of as the degrees of freedom that are used to provide an accurate account of sensory data. The imperative to minimize complexity is manifest in many guises, most commonly referred to in terms of “Occam’s principle,” namely, the simplest of equally accurate explanations is the best (Maisto et al. 2015).

An alternative way of carving up free energy is in terms of “energy” and “entropy” (see the last equality above). These terms inherit, by analogy, from free energy functionals in statistical physics. The energy here is the expected log probability of both the sensory consequences and their causes, under posterior beliefs. The entropy is the uncertainty of those posterior beliefs. The imperative to maximize entropy is commonly referred to in terms of “Jaynes maximum entropy principle” (Jaynes 1957). Heuristically, it refers to the importance of “keeping one’s options open” (Klyubin et al. 2008; van der Meer et al. 2012; Schwartenbeck et al. 2015) or avoiding a commitment to a particular account of how some data were caused. A failure to minimize entropy, and indeed complexity, in statistics is reflected in an over parameterization of the generative model, which leads to overfitting and a failure to generalize to some new (sensory) data. This is a pernicious sort of failure that plagues many applications in machine learning (Hochreiter and Schmidhuber 1997; Lin et al. 2017).

The free energy formulation of degeneracy and redundancy is elemental, and therein lies its significance. Free energy is simply a way to articulate what function means, in relation to degenerate or redundant function. Function here entails maximizing model evidence. This licenses the use of belief updating—with an overtly representational stance—to define degeneracy and redundancy mathematically. In what follows below, we relate these concepts—of entropy and complexity—to degeneracy and redundancy.

### Degeneracy Explained

If we consider the mathematical and statistical definitions of degenerate mappings and redundancy, we see a clear relationship between “complexity” and “redundancy”—and between “entropy” and “degeneracy.” Degenerate solutions, functions, or mappings refer to the nonuniqueness of a solution or a “many-to-one” (injective, nonsurjective) relationship. For example, degenerate eigenvalue solutions in quantum physics mean that there are many linearly independent eigenstates that have the same eigenvalue (Wheeler 1989; Garriga and Vilenkin 2001; Goold et al. 2016). This indeterminacy is seen in the area of ill-posed inverse problems: Returning to the cup lifting example above, not only is there a heuristic degeneracy implied by the use of one hand or the other, the trajectory of the hands during the act of grasping is itself a degenerate or ill-posed problem, known as Bernstein’s problem (Bernstein 1967). In other words, there are an infinite number of ways that “I can move my hand to lift a cup.” This poses a problem when issuing motor commands or predictions to realize a particular functional endpoint. Solving this problem underwrites nearly all Bayesian inference, namely, using (prior) constraints to resolve ill-posed problems, characterized by degenerate mappings between causes and consequences.

To measure this sort of degeneracy, we will associate function with self-evidencing. Then, the essence of degeneracy lies in the “many-to-one” mapping between causal structures in the generative model (i.e., representations or constructs) and the observable outcomes (i.e., sensory data). This means that degeneracy is measured by the number or variety of distinct causes that could produce the same outcome. Mathematically, high degeneracy implies that the posterior probability or belief about causes (i.e., hidden states) will—in the context of a degenerate mapping between causes and consequences—have a large entropy. This is precisely the entropy part of the free energy above. In short, this means that we can associate the entropy of posterior beliefs about the causes of our sensorium with degeneracy. Minimizing free energy therefore requires the maximization of degeneracy, under constraints of energy minimization. This is a ubiquitous conclusion that is found throughout statistics and physics, often referred to as the maximum entropy principle (Jaynes 1957; Banavar et al. 2010; Seifert 2012).

Intuitively, suppose we wanted to infer the causes of some outcome—say “lifting a cup.” Given the observation or goal of “lifting a cup,” there is no implicit information to disambiguate between right- or left-handed lifting movement. Formally, this means our representations (i.e., posterior beliefs) are uniformly distributed over the causes of this “cup lifting” consequence. A completely degenerative mapping from causes to consequences has the highest entropy: here, a 50:50 posterior over two (mutually exclusive) causes. To link this example to lesion studies, if I wanted to use some posterior beliefs to predict what I am going to do, the accompanying structural representations of a right- or left-handed movement are sufficient to produce that outcome. However, if I am unable to represent either cause, I will be unable to realize the outcome.

### Redundancy Explained

In the statistics and neuroscience literature, “redundancy” is the complement of “efficiency” (Barlow 1961; Laughlin 2001; Still et al. 2012; Sengupta et al. 2013). Returning to Bernstein’s problem above, there is one especially efficient trajectory that takes my hand to the cup, with the minimum expenditure of energy. This follows from variational principles of least action, speaking to a unique and maximally efficient movement. The average efficiency can, in the present setting, be associated with redundancy via the equivalence of the principles of maximum efficiency (Barlow 1974; Linsker 1990) and minimum redundancy (Mohan and Morasso 2011). In terms of self-evidencing and free energy minimization, maximizing efficiency corresponds to minimizing the complexity of an inference (possibly about the action that we are currently taking).

Previously, we have defined complexity as the difference between posterior and prior beliefs, that is, beliefs before and after seeing the outcomes (Friston 2009; Kanwal et al. 2017; Da Costa et al. 2020). Therefore, large divergences from prior beliefs to posterior beliefs would incur a greater complexity cost, that is, have a larger redundancy. Taking the example of “lifting a cup,” I can use my right hand, left hand, or both. If a priori I believed I might use either hand—and in witnessing my action, I only used one—the difference between posterior and prior beliefs would be large (high complexity cost). In contrast, if my prior beliefs suggest that I use the hand nearest to the cup, and I use that hand, the difference between posterior and prior beliefs would be small (low complexity cost).

This complexity is another important part of free energy; therefore, minimizing free energy requires a minimization of complexity or redundancy. This minimization manifests in many ways and—under some simplifying assumptions—directly implies the principles of maximum mutual information or the Infomax principle (Linsker 1990). From our perspective, we just need to note that complexity is redundancy and, everything else being the same, redundancy has to be minimized, as dictated by Occam’s principle (Ay 2015; Maisto et al. 2015).

### Degeneracy and Redundancy

This formulation of degeneracy and redundancy has several consequences, some of which are quite revealing. Firstly, degeneracy and redundancy are well-defined measurable quantities, given some outcomes—and a generative model—under ideal (active) Bayesian observer assumptions. Furthermore, they have the same units of measurement (i.e., natural units, if we use natural logarithms of probabilities, bits, if we use binary logarithms). This means degeneracy and complexity are measured in the same currency and can be compared quantitatively. Additionally, they are both attributes of posterior (probabilistic, subpersonal) beliefs. Degeneracy is a statement about the uncertainty (i.e., entropy) of a posterior belief, while complexity is a measure of the relative uncertainty (i.e., relative entropy) of a posterior belief “in relation to a prior belief.” In this sense, redundancy reflects the degree of belief updating, often called “Bayesian surprise” in the visual neurosciences (Itti and Baldi 2009; Sun et al. 2011).

In virtue of the fact that one can measure the expected degeneracy and redundancy (i.e., entropy and complexity) during belief updating, one can associate degeneracy and redundancy with a particular generative model. In turn, this means that degeneracy and redundancy are context-sensitive attributes of a generative model—because belief updating depends upon the data or outcomes at hand. This context sensitivity is important. In other words, what may be redundant in one context or experimental setting may not be redundant in another.

Furthermore, the imperatives to reduce degeneracy and complexity are in opposition. If function is defined in terms of minimizing free energy—or maximizing model evidence—then function entails a “minimization” of redundancy and a “maximization” of degeneracy, under accuracy and energy constraints, respectively. These constraints mean that neither degeneracy nor redundancy is a complete specification of function, when defined in terms of self-evidencing. This means that one cannot talk about minimizing degeneracy or redundancy without knowing the implications for how changes in posterior beliefs affect accuracy and energy. Having said this, there is one important exception. In the absence of sensory data, the free energy reduces to redundancy, because the accuracy term disappears, leaving only complexity. This means that optimizing a generative model offline (e.g., during introspection or sleep) affords the opportunity to minimize redundancy (Hinton et al. 1995; Hobson and Friston 2012).

Crucially, one cannot change degeneracy without changing redundancy—and vice versa. This follows naturally from the free energy formulation above, which means that redundancy equals “cost” minus degeneracy, where cost is the negative expected value of the inferred state of affairs—and value is the logarithm of prior preferences (Friston et al. 2015):

$$ \underset{\mathrm{redundancy}}{\underbrace{D_{KL}\left[Q(s)\Big\Vert P(s)\right]}}=\underset{\mathrm{cost}}{\underbrace{{\mathbb{E}}_Q\left[-\ln P(s)\right]}}-\underset{\mathrm{degeneracy}}{\underbrace{{\mathbb{E}}_Q\Big[-\ln Q(s)}}\Big] $$

In other words, redundancy can be interpreted as a cost that is offset by degeneracy, highlighting the opposing roles of redundancy and degeneracy. This relationship also speaks to the pre-eminent role of prior beliefs in defining the relationship between degeneracy and redundancy—and their contributions to evidence or marginal likelihood. These prior beliefs are the quantities that are optimized during learning, for example, experience-dependent plasticity (Gershman and Niv 2010; Tervo et al. 2016; Testolin and Zorzi 2016; Gershman 2017) and, importantly, changes to the structure of the generative model due to lesions. In short, changes to the structure can always be articulated in terms of changes to a prior (Friston and Penny 2011). Thus, the changes to the priors necessarily change the posterior (following belief updating), and changes in the two necessarily mean a change in redundancy and degeneracy.

From a practical perspective, given these definitions, it is clear that to quantify degeneracy and redundancy, one needs the entire belief structure—and belief updating—that accompanies experience-dependent learning. In turn, this means that it is necessary to measure the distributed aspects of probabilistic representations in the brain. Put another way, redundancy and degeneracy cannot be localized to a particular representation or neuronal system; they are properties of distributed representations that underwrite belief updating throughout (hierarchical) generative models or cortical structures. By distributed representations we are referring to how sensory features of observed outcomes are being encoded neuronally. This may have important implications for understanding the impact of lesions—as scored through changes in degeneracy and redundancy. Note that in this quantitative treatment, degeneracy and redundancy are attributes of a function, namely, some perceptual recognition or overt action. As noted above, this kind of degeneracy (and redundancy) can be context-sensitive and therefore cannot be inferred from anatomical connections alone—it can only be inferred from the functional message passing along these connections. In this sense, the empirical measurement of degeneracy and redundancy necessarily relies upon some form of functional neuroimaging or electrophysiology.

In the remainder of this paper, we unpack some of the above points and pursue their construct validation using simulations of active inference, where we know exactly the form of belief updating and can measure degeneracy and redundancy. Our aim is to illustrate the correspondence between these mathematical quantities and the use of these terms in the context of lesion studies.

## Simulations of Word Repetition

In this section, our objective was to illustrate how learning or experience-dependent plasticity in neuronal structures (i.e., the brain’s generative model) produces changes in degeneracy and redundancy. For this, we chose a canonical paradigm in the neuropsychology of language, namely, word repetition (Burton et al. 2001; Hope et al. 2014). The first step was to specify a generative model and active inference scheme that is apt for simulating this paradigm. We used a (Markov decision process) generative model of discrete outcomes that are caused by discrete hidden states: described extensively in Friston, FitzGerald, et al. (2017a), Friston, Lin, et al. (2017b). For technically minded readers, we have included a detailed description of the generative model and accompanying belief updates in the Appendix. These equations are a bit involved; however, the generative model on which they are based is very general—and can be applied in most settings— where outcomes and their causes can be expressed in terms of distinct (i.e., discrete) states.

Through our simulations, we evaluated the minimization of redundancy, using a formulation of structure learning. In other words, after some suitable experience with—and learning of—a word repetition task, we withheld sensory information and adjusted the parameters of the generative model (i.e., connection strengths) to minimize complexity and thereby eliminate redundant parameters. We then repeated the paradigm to quantify the effects of complexity and degeneracy. We anticipated that this targeted elimination of redundant parameters would selectively suppress redundancy in relation to degeneracy. In the subsequent section, we use a similar manipulation but reduce nonspurious connectivity to simulate synthetic lesions.

### The Generative Model

**Figure 1:**
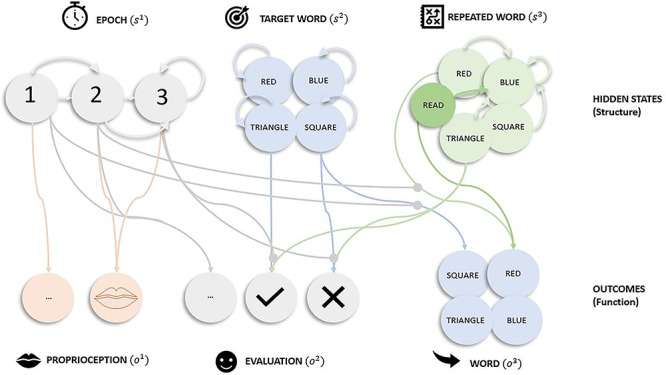
Generative model. Graphical representation of the generative model for word repetition. There are three (hidden) state factors, epoch, target word, and repeated word, and three outcome modalities, proprioception, evaluation, and audition. The hidden state factors had the following levels (i.e., possible alternative states). Epoch (three levels) indexes the phase of the trial. During the first epoch, the target word is heard. The second epoch involves repeating the word. The third phase elicits a positive evaluation, if the repeated word matches the target word and a negative evaluation otherwise. The repeated word factor includes the words that our (synthetic) subject can choose to say (five levels). Note the deliberate inclusion of the spurious word “read” (darker green). The target word factor (four levels) lists the words the experimenter can ask the participant to repeat. The lines from states to outcomes represent the likelihood mapping, and lines mapping states within a factor represent allowable state transitions. For clarity, we have highlighted likelihoods and transition probabilities that are conserved over state factors and outcome modalities. For example, the “audition” likelihood mapping the target word (*square*) to audition (*square*) is shown for epoch 1, but similar mappings apply, when mapping between “blue” and “blue.” It is important to note the spurious repeated word “read” (darker green) is treated the same as other levels for this particular factor; that is, for the audition likelihood, it maps directly to audition modality “red” for epoch 2. One (out of a total of five) example transition probability is highlighted for the repeated word; that is, the transition is always to blue, regardless of previously spoken word (red, read, triangle, square, or blue). This transition represents the choice to say “blue.” Similar mappings are applied when choosing to say “triangle,” regardless of the previous word. Alternative actions then correspond to alternative choices of transition probability.

The paradigm used to illustrate the differences between degeneracy and redundancy was a word repetition task: The subject is presented with a single word (e.g., triangle, red, etc.), at each trial, and asked to repeat it. If the agent repeats the word correctly, they are given a positive evaluation (and negative otherwise). The generative model—comprising appropriate probability distributions—was deliberately minimal to illustrate the role of redundancy and degeneracy (Fig. 1). The (categorical) probability distributions were based on an empirical understanding of how subjects respond in a word repetition paradigm (Hope et al. 2014). Specifically, it is an expression of how experimental stimuli are generated during an experiment (conditioned upon subject responses). We assume that subjects adopt similar models to mirror this process. This model can be plausibly scaled to account for larger lexical content (i.e., words) or equipped with lower hierarchical levels to model articulation.

The generative model has three (hidden) state factors, “epoch, target word, and repeated word,” along with three outcome modalities, “proprioception, evaluation, and audition.” The “epoch” factor covers stages of the trial: listening to target word (i.e., epoch 1), repeating the target word (i.e., epoch 2), and receiving performance evaluation (i.e., epoch 3). The “target word” factor has states corresponding to a heard word: red, blue, triangle, or square. The “repeated word” factor’s states covered what should be repeated: blue, triangle, square, red, and a spurious level “read” (to model redundancy). The level “read”—past tense of read and pronounced in the same way as red—is spurious within our model specification. This is because despite having the same phonetic form as “red,” it denotes something quite different (study instead of a color). The spurious level “read” has been included to introduce some redundancy within the belief space. In terms of outcome modalities, the “proprioception” outcome reports whether my mouth moved or not. The “audition” outcome reports the current (spoken or heard) word: red, blue, triangle, or square. The “evaluation” outcome represents the positive or negative response received (only provided at the third epoch). In Figure 1, the lines represent plausible connections (and their absence reflects implausible connections), with the arrow denoting direction. For example, the line mapping hidden state epoch “1” to outcome modality proprioception “…” suggests that “…” is only plausible at epoch “1,” but not “2” or “3.” Similarly, the line for hidden state target word “blue” to itself reflects that level “blue” can only transition to itself and no other word, throughout the trial.

The likelihood (**A**)—mapping between states (i.e., causes or structures) and outcomes (i.e., consequences or functions)—is represented by the lines connecting states to outcomes in Figure 1. When inversion of a model is formulated in terms of neuronal message passing, these connections take the form of extrinsic connections (i.e., between brain regions or neuronal populations encoding posterior expectations). Each sensory (outcome) modality is associated with its own likelihood. The “proprioceptive” likelihood depends on the “epoch” factor; if I believe it is epoch 1 and I am listening to the target word, then my mouth is not moving. If I believe it is epoch 2 and I am repeating the target word, then my mouth is moving. The “audition” likelihood depends on either “repeated word” (for epoch 2) or “target word” (for epoch 1) factors. Which of these is responsible for generating auditory input depends on the epoch in play?: For example, in epoch 1, the “audition” likelihood maps the target word (square) to auditory input (square). For state “red/read,” both the original and spurious are mapped to the same auditory outcome (“red”) for when I am speaking, as shown in Figure 1. The likelihood is defined as a one-to-one mapping between the “target word” and “audition,” if I believe I am at epoch 1 of the trial. Conversely, if I believe I am at epoch 2 or 3, there is a one-to-one mapping between “repeated word” and “audition.” The “evaluation” likelihood depends on all the hidden states. Positive evaluation is given at epoch 3, if I am repeating the previously heard word correctly. For example, if I am repeating triangle—after hearing triangle (resp. square)—I will get positive (resp. N. negative) evaluation. The likelihood is defined as mapping to 1) neutral feedback—regardless of “target” and “repeated word”—if the epoch is 1 or 2; 2) positive feedback, if the repeated and target word match at epoch 3; and 3) negative otherwise.

The transition matrices, **B**—transitions among the hidden states encoding prior beliefs about trajectories or narratives—are represented by lines modeling transitions among states within each factor in Figure 1. When interpreted as message passing between neural populations, these denote intrinsic connections (i.e., connectivity within brain regions or neuronal populations encoding posterior expectations) as they map from and to the same set of states. For the “epoch” factor, the transitions are from 1 to 2 and 2 to 3 with 3 being an absorbing state (i.e., the final epoch is of the third epoch type). For the “repeated word” factor, there are five possible transitions. These involve transitions to a specific word, where the word depends upon which action is selected. An example transition would be that when I choose to say “blue,” regardless of previous word (red, triangle, etc.), I transition to blue (highlighted in Fig. 1). The transition matrix for the “target word” is an identity matrix. This means the target word stays the same over all epochs.

The likelihood and transition matrices outlined above can themselves be learned over multiple trials of the word repetition task, resulting in changes in degeneracy and redundancy associated with belief updating. This rests upon accumulation of Dirichlet parameters that act as “pseudocounts” and encode synaptic connection strengths. The more often a given pairing (of state and outcome or past and present state) is observed, the greater the number of counts attributed to that pairing. By dividing the number of counts for each by their total, we arrive at the new (learned) probability distributions. It is worth emphasizing two aspects of this accumulative process. The first is that it closely resembles Hebbian plasticity, where synaptic efficacy is incremented upon the simultaneous firing of a pre- and postsynaptic neuron. The second is that this plastic potential depends upon the number of Dirichlet counts assumed at first exposure to the task. This number may be thought of as quantifying the confidence in prior beliefs about these conditional probability distributions and the resistance to moving away from these priors. To ensure learning the initial Dirichlet concentration parameters, of both the likelihood and transition prior distributions, were set to 1 for plausible and 0.5 for implausible elements. During learning, we hoped to demonstrate an overall reduction in redundancy—in relation to accuracy—and an increase in degeneracy, in relation to energy. This allows us to explicitly represent how redundancy may evolve over time within a system; our model learns to understand its environment allowing for redundancy to go down (with appropriate evidence accumulation) until it plateaus. Please see Friston et al. (2016) for a discussion of learning under these active inference schemes.

The simulated subject was equipped with strong preferences (measured in nats, i.e., natural logarithm) for receiving positive evaluation (0.5 nats). Additionally, the subject was allowed to choose from a set of five different deep policies (sequences of actions), each of which is a different permutation of how (controlled) state transitions might play out. The prior beliefs about the initial states were initialized to 10 for all “repeated” and “target word” levels, epoch 1 for the “epoch” factor and zero otherwise. The precision over action selection (α) was specified as a relatively high level of 16 (Schwartenbeck et al. 2015). Henceforth, any mention of “subjects” refers specifically to simulated active inference models, based on random initialization.

### Evaluating Redundancy

We have associated redundancy with complexity, namely, the difference between posterior beliefs and prior beliefs about hidden states generating outcomes. Using this definition, we can measure redundancy (via complexity), given some outcomes and specified generative model, under ideal Bayesian observer assumptions. We start with a simple setup. The generative model—with the spurious level (“read”) as described above—was used to simulate 500 trials for 50 different subjects (based on random initialization seeds). For each trial the subject had to repeat one of the four possible words (i.e., “red,” “square,” “triangle,” or “blue”).

**Figure 2:**
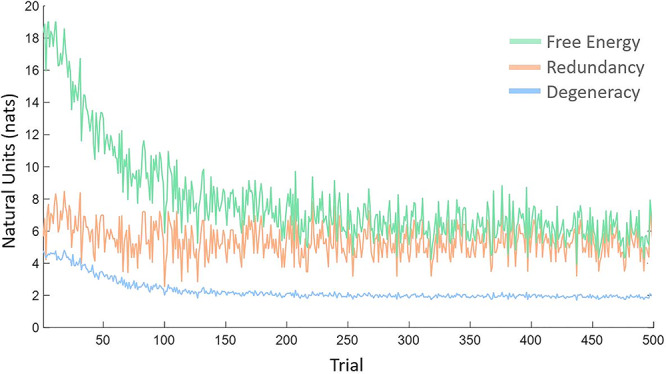
Learning-dependent changes in degeneracy and redundancy. This figure plots trial-specific estimates of free energy, degeneracy (i.e., posterior entropy), and redundancy (i.e., complexity)—averaged over all hidden factors, epochs, and subjects as a function of exposure to the word repetition task (over 500 trials) for the model with the spurious specification.

To illustrate the effects of learning on degeneracy and redundancy, we computed the free energy, posterior entropy (i.e., degeneracy) and complexity (i.e., redundancy) for each trial (averaged over epochs, hidden factors, and subjects). The results are shown in Figure 2 as a function of trials. As anticipated, there is a gradual reduction in free energy, that is, a large increase in model evidence due to experience-dependent learning. This is accompanied by a reduction in redundancy and a small reduction in degeneracy. In other words, skill acquisition or structural learning under this word repetition task increases model evidence by reducing redundancy. In what follows, we turn to the effect of changing connections, not by experience-dependent learning but by selectively removing certain connections. To examine this, we precluded (further) learning by preventing any further updates to the model parameters (i.e., connectivity).

**Figure 3:**
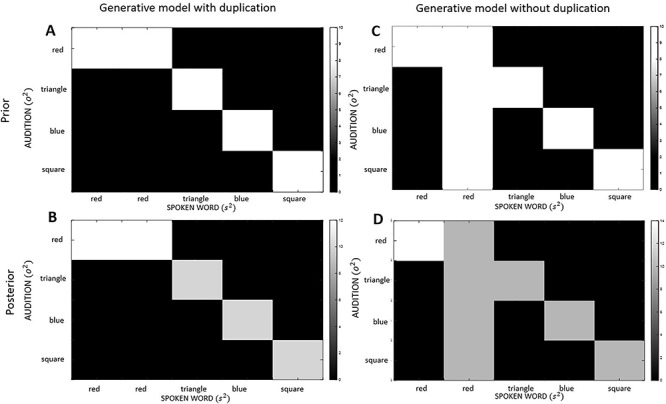
Dirichlet concentration parameters. This graphic shows the Dirichlet concentration parameters (that encode synaptic connection strengths) for the “audition” likelihood, that is, for “repeated” word factor mapping to “audition” with respect to “target word” triangle, at epoch 3. The scale goes from white (high concentration) to black (low concentration), and gray indicates gradations between these. The top row represents the prior beliefs for both the generative model with (*A*) and without (*C*) the spurious level: The key difference to note is the prior beliefs for the model without the spurious level (i.e., “read”) are completely uniform. The bottom row represents the posterior beliefs, after learning, for the model with (*B*) and without (*D*) the spurious level: The key difference to note here is that the posterior beliefs for the model with spuriousness are less precise due to two plausible options, highlighted by the white in the first two columns (the first “red” and second “read” in panel *B*). In contrast, the posterior beliefs for the first “red” in the model—without spurious connections—are very precise due to only one plausible option (i.e., first “red” in panel *D*).

We next examined the effects of removing redundant model parameters or connections. In brief, we simulated structure learning by removing the spurious hidden state (i.e., “read”). This was implemented by setting the Dirichlet concentration to the same value (i.e., 10), for all the likelihood mappings associated with the repeated word, “read.” This means the model makes imprecise predictions for all sensory outcomes that are generated by “read.” In effect, this disconnects the representations of a hidden state from representations of sensory outcomes. An example of the difference in Dirichlet parameters between the spurious and nonspurious generative model for the “repeated word” factor mapping to “audition” is shown in the top row of Figure 3 (with respect to target word “red,” at epoch 3).

**Figure 4:**
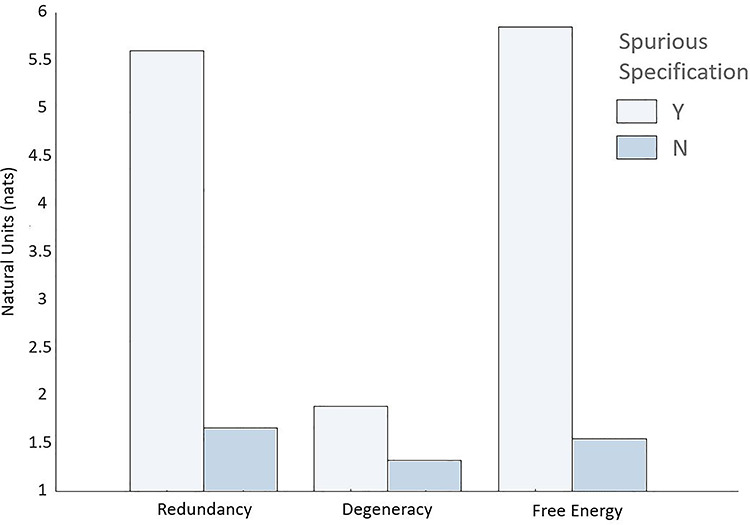
The effects of structure learning. This figure reproduces the data in the first two rows of Table 1. It highlights the effects of removing redundant connections (i.e., parameters) of the generative model on redundancy, degeneracy, and free energy. The key point to take from these results is that selective removal of redundant connections decreases free energy (increases model evidence) driven by the decrease in redundancy.

We reran the experimental paradigm in the absence of redundant connections (i.e., disconnected the spurious level)—for 50 simulated subjects across 10 trials. Here the Dirichlet concentration—excluding the likelihood mappings associated with the spurious repeated word “read”—was parameterized using the postlearning (i.e., after the 500 trials) probability distribution from the prior simulations. We then measured the total redundancy and degeneracy (averaged over epochs, trials, hidden factors, and subjects). The results are shown in the first two rows of Table 1, eight simulations with (Y) and without (N) spurious connections. For ease of visualization, the free energy, redundancy, and degeneracy are also shown as bar plots in Figure 4, with and without the removal of spurious connections.

**Table 1:** Free energy and its components

Group type	Spurious	Trials per agent	Free energy	Redundancy	Degeneracy	Cost	Energy	Accuracy
			*F*	${D}_{KL}\Big[Q\Big.(s)\Big||P(s)\Big]$	${\mathbb{E}}_Q\Big[-\ln Q(s)\Big]$	${\mathbb{E}}_Q\Big[-\ln P(s)\Big]$	${\mathbb{E}}_Q\Big[-\ln P\Big(s,o\Big)\Big]$	${\mathbb{E}}_Q\Big[\Big.\ln P\Big(o|s\Big)\Big]$
Control	Y	500	5.803	5.631	1.981	7.611	7.784	−0.173
Control	N	10	1.831	1.752	1.340	3.092	3.172	−0.080
B	Y	10	6.871	6.871	3.590	10.461	10.461	0.000
B	N	10	2.805	2.742	2.986	5.728	5.790	−0.063
A	Y	10	13.252	5.800	2.028	7.828	15.280	−7.452
A	N	10	16.324	8.717	1.561	10.279	17.886	−7.607
A and B	Y	10	14.876	7.075	4.825	11.900	19.700	−7.801
A and B	N	10	19.942	11.918	4.303	16.221	24.245	−8.024

This table summarizes the simulations in terms of the respective free energy, redundancy (complexity), accuracy, degeneracy (entropy), energy, and cost measurements. These are measured in natural units. There are four sorts of simulations: control (with no lesions), intrinsic lesions, extrinsic lesions, and both intrinsic and extrinsic lesions. The simulations were repeated with (Y) or without (N) spurious representations. The values are averages calculated for all the state factors across each epoch and simulated subjects. Note that in order to appropriately simulate learning, 500 trials were simulated per agent for the control group with the spurious level, relative to the 10 trials simulated for all other groups.

**Figure 5:**
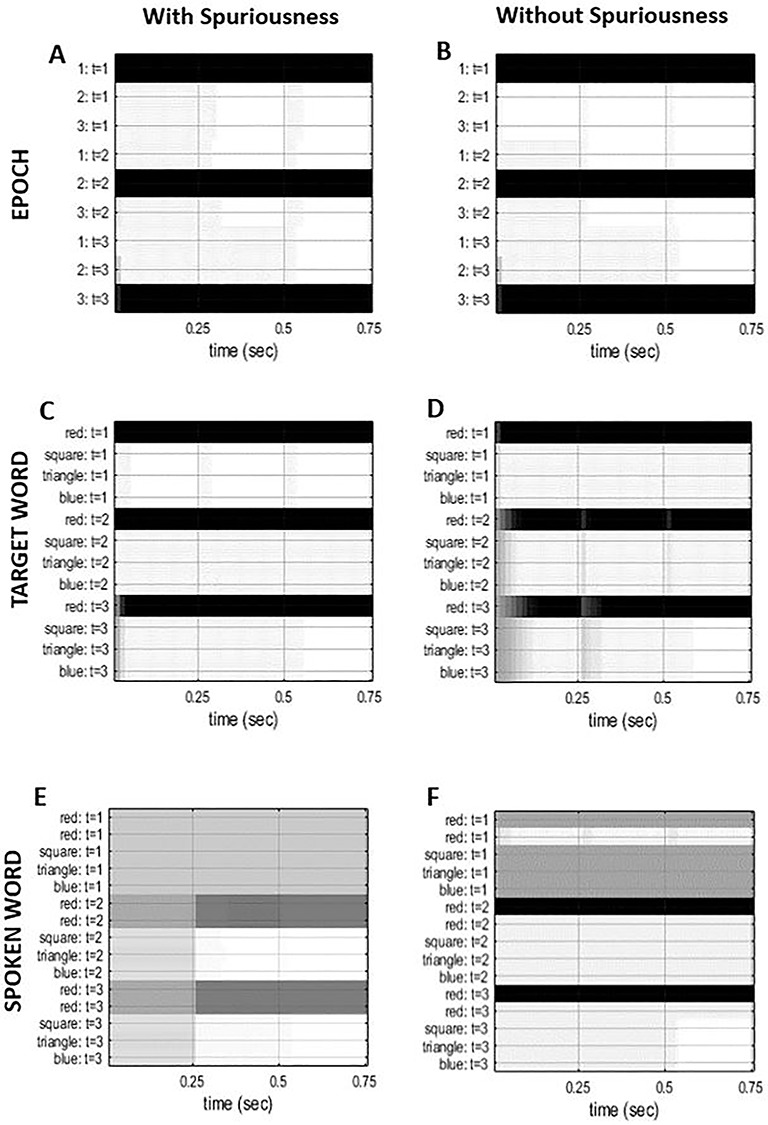
Belief updating for model with (left panels) and without (right panels) the spurious level. Each panel reports belief updating over three epochs of a single trial, for the factors (*epoch, target word, repeated word*) when repeating the word red. The *x*-axis represents time in seconds (divided into 3 epochs), and the y-axis represents posterior expectations about each of the associated states at different epochs (in the past or future). For example, for the spoken (repeated) word factor (E, F), there are 5 states, and a total of 5 × 3 (states times epochs) posterior expectations – similarly, for the epoch factor (A, B), there are three levels, and total of 3 × 3 expectations. For epoch *target word* factor (C, D), there are 4 states, and total of 4 × 3 posterior expectations. White is an expected probability of zero, black of one, and grey indicates gradations between these. For example, the first five rows in panel E, correspond to expectations about the *repeated word*, in terms of five alternatives for the first epoch. The second five rows are the equivalent expectations for the second epoch. This means that at the beginning of the trial the second five rows express beliefs about the future; namely, the next epoch. However, later in time, these beliefs refer to the past; i.e., beliefs currently held about the first epoch. This aspect of (deep temporal) inference is effectively an implementation of working memory that enables our subject to remember what she has heard—and accumulate evidence for the target word that is subsequently articulated. Note that most beliefs persist through time (along the *x*-axis). For example, the target word reveals itself almost immediately in panel C and this prospective belief is propagated into the future. Note further that the belief updating is similar across the two generative models, except for the *repeated word* factor—where even at the first epoch–the subject believes that the spurious state (“read”) is an implausible hypothesis, for the present and the future

The targeted elimination of spurious (connectivity) parameters selectively reduced redundancy in relation to degeneracy: The decrease in redundancy (of 3.88 nats)—when comparing the spurious and nonspurious structures—was driven by cost (reduced by 4.52 nats), as opposed to degeneracy (reduced by 0.64 nats). This is consistent with the interpretation of redundancy as a cost that is offset by degeneracy. It also demonstrates how prior beliefs define the relationship between degeneracy and redundancy: Differences in priors produce differences in posteriors—as exemplified by the second row of Figure 3 (posterior beliefs in the spurious model are less precise, compared to posterior beliefs in the model without spurious representation). In short, changes in priors and posteriors necessarily entail changes in redundancy and degeneracy. Furthermore, minimizing redundancy by changing posterior beliefs has a direct impact on accuracy of the generative model, here, an increase in accuracy of 0.09 nats. However, as detailed below, this has little impact on behavior (postlearning), due to the very similar belief updating under the two models (Fig. 5).

## In Silico Lesion Studies

This section looks more closely at the effects of lesions on function, as scored by changes in free energy, degeneracy, and redundancy—and accompanying neuronal and behavioral (i.e., accuracy) responses. Lesions were simulated by changing the priors of the generative model (i.e., Dirichlet concentration parameters) in the same way as the redundant connections were removed in the previous section. However, here, we are removing the “wrong kind” of connections to simulate a lesion—as opposed to removing the “right kind” of (redundant) connections to simulate structure learning. Our aim was to demonstrate a profound “increase” in redundancy and concomitant accuracy deficits, particularly with dual (synthetic) lesions.

Here a “lesion” corresponds to removing synaptic connections that encode the likelihood and prior transition probabilities (in the **A** and **B** matrices). Given that the likelihood mapping couples adjacent levels of hierarchical generative models, we can associate the **A** matrix with the extrinsic (between-region) connectivity (i.e., adjacency) matrix describing the anatomical connections between levels in cortical hierarchies. In other words, we assume that the **A** matrix embodies extrinsic connectivity. Conversely, because the probability transition matrices are local to any given hierarchical level, we associated the corresponding Dirichlet parameters with intrinsic (within-region) connectivity. These structural assumptions mean that we can regard lesions to the **A** matrix as reproducing the kind of disconnections that would follow a stroke that impinges upon white matter tracts. Conversely, any neurodegenerative processes that involve a loss of synaptic connections can be associated with lesions to the probability transitions or **B** matrices. Clearly, lesions might entail the changes to both **A** and **B** connectivity parameters. To motivate the neural anatomical distinction between the parameters of the likelihood and transition probabilities, we now briefly consider the functional neuroanatomy of word repetition.

### Computational Architecture of Word Repetition

To associate in silico lesions to neurological disorders and neurodegenerative process, the belief updates have to be associated with neuronal circuits. At the level of canonical microcircuits, there is an established process theory that allows us to map message passing on to intrinsic connectivity within gray matter: for details see (Bastos et al. 2012; Shipp 2016a, 2016b; Friston, FitzGerald, et al. 2017a; Friston, Parr, et al. 2017c). However, to associate the simulated belief updating with functional neuroanatomy, it would be necessary to assign different neuronal populations to particular brain structures. These are hypothesis-generating assignments and allow us to retain the potential to speak to neuroimaging studies in future work. In the discussion, we consider how this could be done using dynamic causal modeling—based upon the kinds of simulations reported below. At present, we will briefly consider the form of the cortical hierarchies implied by the generative model in Figure 1.

**Figure 6:**
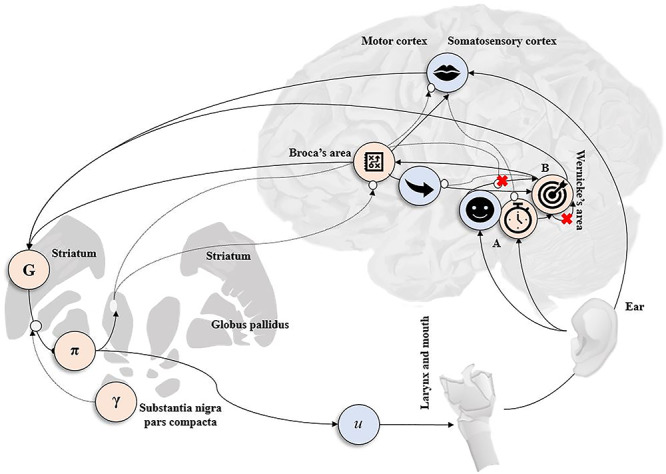
The computational architecture for word repetition. This graphic illustrates idealized message passing—as defined by the generative model—for word repetition. The outcomes (blue with icons), hidden states (pink with icons), and model variables (G, expected free energy; π, expected policies; γ, expected precision; and u, action) are assigned to neuronal populations. The icons correspond to the hidden factors and outcome modalities in Figure 1. The black arrows denote message passing, and the white circular dots indicate a modulatory weighting between neuronal populations. The smiley face icon (in the auditory cortex) represents processing of auditory inputs from the ear. The target icon (in Wernicke’s area) represents the target word (i.e., speech sounds that are recognized and need to be repeated), and stopwatch icon (in the hippocampus) is responsible for the temporal representation of each trial. The cross-box icon (in Broca’s area) represents an internal model of the intended speech that drives motor output (in the motor cortex controlling the mouth and larynx) and predicts how motor activity will change proprioception in the postcentral gyrus (mouth icon) and auditory processing in the auditory cortex (smiley icon). The red crosses represent the site of in silico lesions—A (extrinsic connections) represents a disconnection from the representations of evaluations, and B (intrinsic connections) represents target word transitions. Expectations about policies per se and the precision of these beliefs have been attributed to striatum and substantia nigra pars compacta areas to indicate a putative role for dopamine in encoding precision (Friston, FitzGerald et al. 2017a).

Having specified the generative model, standard message passing schemes effectively define the requisite computational anatomy in terms of nodes (e.g., neuronal populations) and edges (e.g., neuronal connections) along which messages (e.g., neuronal firing) are passed (Friston, Parr, et al. 2017c). There are certain aspects of this computational anatomy that can be mapped onto the functional anatomy in the brain, particularly, those components involved in policy selection (Friston et al. 2014; Friston, FitzGerald, et al. 2017a). Figure 6 presents a probabilistic graphical model (PGM) to illustrate the computational architecture (i.e., graph) implied by our generative model. Nodes represent hidden states (see Fig. 1), and the edges denote conditional dependencies. These edges contain the parameters of the generative model and correspond to (intrinsic and extrinsic) anatomical connections in the brain. To emphasize the distinction between our disconnections (i.e., lesions) of extrinsic (A) and intrinsic (B) connectivity, we have equipped this PGM with recurrent (i.e., self) connections that stand in for the probability transition parameters of the generative model. The red crosses indicate where we have, effectively, lesioned a particular pathway.

The assignment of nodes in this graphical model to anatomical regions is speculative—as with all neuronal process theories that attend free energy minimization (Gu et al. 2018). However, it illustrates that message passing between neuronal representations, under certain assumptions, can be plausibly associated with extrinsic connections in the brain. Specifically, under the assumption that neuronal populations encode the statistics of posterior probabilities, transition probabilities can be regarded as being parameterized by neuronal connections (Friston et al. 2016). This allows one to consider functional deficits following synthetic lesions in relation to axonal disconnections or degenerative neuronal loss. For example, see (Parr and Friston 2017) and (Parr, Benrimoh, et al. 2018), respectively.

To simulate lesions, we selectively reduced the strength of the strongest connections—and increased the strength of the weakest connections—via a decrease in the precision hyperparameter. Mathematically, this decreases the overall precision of the associated likelihood or prior distribution (over outcomes or states, respectively). We were particularly interested in the cumulative effect of increasing the extent of (extrinsic (**A**) or intrinsic (**B**) or both) lesions on function, as scored by degeneracy and redundancy—and the accompanying behavioral and neuronal responses.

We examined the interaction between both kinds of (intrinsic and extrinsic) lesions, noting that typical neurological disorders probably involve both. Specifically, to mimic the kind of disconnection that would follow a stroke, we lesioned the mapping between the *evaluation* outcome modality and all hidden states (Fig. 4: black circle with smiley face icon). Secondly, we lesioned the “target word” **B** transition matrix, with and without lesions to the **A** matrix. This limits the subject’s ability to track the target word, resulting in perceptual impairment and the production of incorrect words. Our aim was to demonstrate an increase in redundancy following lesions that was not compensated for by an increase in degeneracy. Furthermore, we hoped to demonstrate a nonlinear effect on accuracy, namely, that the cumulative effect of introducing more lesions would be superadditive, producing performance deficits, with a sufficient lesion load. This speaks to the notion that particular combinations of distributed lesions are necessary to produce a deficit—a hallmark of a degenerate functional anatomy.

### Quantitative Evaluations

**Figure 7:**
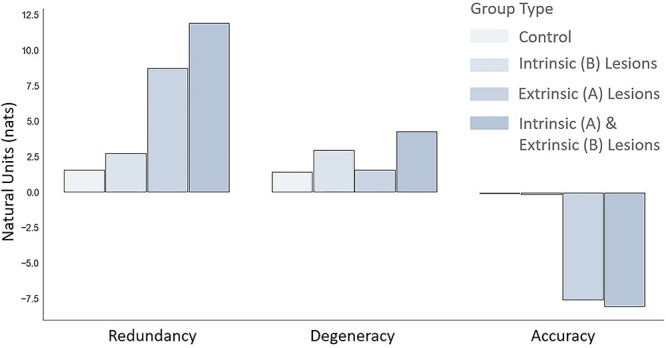
Effects of lesions. This bar chart reproduces some of the data in Table 1 to highlight the effects of combined (extrinsic and intrinsic) disconnections on redundancy, degeneracy, and accuracy. Note that these measures are directly interpretable in terms of their statistical evidence. By convention, a difference in log evidence (i.e., a log Bayes factor) of three is considered strong evidence (corresponding to a log odds ratio of 20:1) (Kass and Raftery 1995). Please see Figure 8 for a more comprehensive analysis of (behavioral and statistical) accuracy.

We start with lesions to the intrinsic connections (in the **B** matrix): The strength of the largest connections was reduced, and strength of the smallest connection was increased via a decrease in the precision hyperparameter from 1 to 0.5. The lesion is performed on the generative model that has already learnt the appropriate probability distributions via structure learning (see previous section). We then simulated the word repetition paradigm using the lesioned model—with and without spurious representations from the previous section—for 50 different subjects across 10 trials. From this, we measured the total redundancy and degeneracy: The results are shown in the third and fourth row of Table 1 and Fig. 7. The lesion increased degeneracy by ~1.6 nats, for both spurious and nonspurious models. The effects of these lesions on redundancy were of a slightly lower magnitude at ~1 nats.

To lesion the extrinsic connections mediating messages between regions (i.e., the **A** matrix), the strength of the largest connections was reduced, and the strength of the smallest connection was increased via a decrease in the precision hyperparameter for **A** from 1 to 0.4. As above, the lesioned generative model with and without spuriousness was simulated for 50 different subjects across 10 trials. The results are shown in the fifth and sixth row of Table 1 and illustrated in Figure 7. The key result here is a profound increase in redundancy that is not matched by an accompanying increase in degeneracy for the model without the spurious level. In summary, lesioning the intrinsic connections had a slightly greater effect on degeneracy than redundancy, whereas lesioning the extrinsic connections had a much greater effect on redundancy (which reflects an increase in the degree of belief updating required).

Finally, we implemented a combination of distributed lesions—on both **A** and **B** matrices—via changes in the connectivity parameters as specified above. The results are shown in the last two rows of Table 1 and illustrated in Figure 7. Two things are immediately evident from these results: Distributed lesions to both extrinsic and intrinsic lesions produced the highest redundancy and degeneracy and the worst performing models in relation to statistical accuracy (a proxy for performance). Both effects have a clear explanation. Uncertain posterior beliefs about causes—in the context of a degenerate mapping between causes and consequences—result in higher degeneracy. The low levels of accuracy are a consequence of these less confident beliefs about what is causing outcomes.

**Figure 8:**
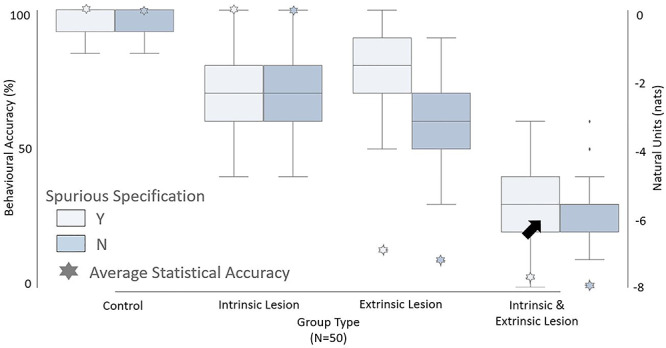
Simulated performance accuracy. The left *y*-axis represents the behavioral accuracy as measured by percentage of correct responses, the right *y*-axis represents natural units, and the *x*-axis represents the different groups. Each group is separated based on model specifications, with (Y) or without (N) the spurious level. For the control group with spurious specification, we report the performance accuracy postlearning (i.e., the last 10 trials). The stars represent the average statistical accuracy for each group (see Table 1 for details). Both control and intrinsic (B) lesion groups perform well with an average statistical accuracy of 0.00 nats and an average behavioral accuracy of 70–100%. The group with extrinsic (A) lesion performs reasonably well in terms of average behavioral accuracy (~70%) despite significantly lower average statistical accuracy at ~7.5 nats (Fig. 7). However, the group with lesions to both extrinsic (A) and intrinsic (B) connections performs badly, with the distribution spread out anywhere between 0% and 60%, an average behavioral performance of ~26% (black arrow) despite a statistical accuracy (~7.9 nats) that is comparable to that of the A lesion group (Fig. 7).

Finally, we evaluated behavioral responses—as measured by behavioral and statistical accuracy—for our stimulated groups: each with 50 patients, performing 10 trials (Fig. 8). Here, behavioral accuracy is measured by percentage of correct responses over the course of the trial. Our aim here was to test for superadditive effects of behavioral performance in a degenerate architecture. The behavioral accuracy for the control, **B** lesion and **A** lesion groups, was good (mean: ~70%) across both model specifications (see Fig. 8) even though the A lesion group had much lower statistical accuracy (mean: −7.5 nats, see Fig. 7). However, lesions to both the extrinsic and intrinsic connections (**A** and **B**) had much worse performance (mean behavioral accuracy of ~26%, Fig. 8) than lesions to the extrinsic lesions alone, despite comparable statistical accuracy (Fig. 7). This is because of the increase in degeneracy associated with uncertain mappings between causes and outcomes. From a lesion-deficit study perspective, the behavioral performance reflects the superadditive effect of lesions on functional deficits, in terms of a “many-to-one” structure–function mapping, such that when multiple pathways are lesioned, it is difficult to perform the task accurately. This is despite only incremental effects on statistical accuracy, redundancy, and degeneracy.

In summary, using numerical analyses, we have illustrated how degeneracy differs from redundancy—and how these measures of processing respond differentially to changes in the structure or connectivity of a generative model. Specifically, we have seen that decreasing the precision of likelihood mappings (by lesioning extrinsic connections) can have profound effects on redundancy. Heuristically, if I am unable to represent the causes of my sensations, I will be unable to realize preferred outcomes and will evince a functional deficit. This is not the case, for when I can use posterior beliefs to predict what I am going to do; the accompanying structural representations of “epoch,” “repeated,” and “target” word are sufficient to produce that outcome—for this particular paradigm and accompanying generative model. In the final section, we consider the physiological correlates of belief updating that would enable some of the predictions entailed by this formulation of degeneracy and redundancy to be assessed empirically.

### Physiological Predictions

To characterize the effect of lesions on belief updating, we examined the (synthetic) subject’s responses to unexpected outcomes; i.e., a “wrong” evaluation. This allowed us to simulate mismatch negativity (MMN) (Garrido et al. 2009; Morlet and Fischer 2014) or P300 (Donchin and Coles 1988) waveform differences. We report these simulations to show how the message passing scheme can be used to make predictions about empirical neuronal responses. Our specific hypothesis was that a sufficient reduction in degeneracy (i.e., increasing posterior uncertainty) would attenuate responses to violations that mediate belief updating. Put simply, with imprecise likelihood and prior mappings, patients may find everything surprising resulting in no difference between expected and unexpected outcomes.

The violation paradigm was modeled, using the nonspurious specification, by giving each synthetic subject the evaluation “wrong” at epoch 3 of the trial. Apart from this, everything else remained the same. This allowed us to stimulate an oddball paradigm in which the subject, expecting an evaluation of “correct,” was now exposed to an unexpected evaluation. Any differences in responses to the standard and deviances can be considered as a simulated MMN (Näätänen et al. 2007). Mismatch responses were simulated for the four different groups: control, extrinsic (**B**) lesion, intrinsic (**A**) lesion, and both (**A** and **B**) lesions. For each simulation, we simulated two trials: one where the synthetic subject heard the correct evaluation and a second where it was given the unexpected “wrong” evaluation. The subject’s internal state, for the “target word,” was always “red” (only one plausible representation).

**Figure 9:**
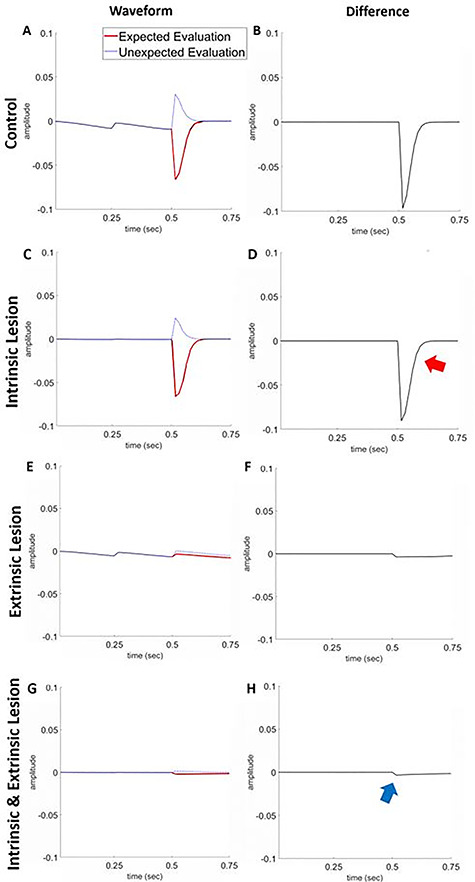
Simulated mismatch negativity responses. The left panel reports simulated electrophysiological responses of a neuronal population responding to the correct evaluation at epoch 3 with (red lines) and without (blue lines) the expected outcome. The differences between these two responses are shown in the right panel and can be read in the spirit of a mismatch negativity or P300 waveform difference. Each row is for a different group: control (*A*–*B*), intrinsic lesion (*C*–*D*), extrinsic lesion (*E*–*F*), and both intrinsic and extrinsic lesions (*G*–*H*). The *y*-axis is the response to stimuli in arbitrary units. The *x*-axis represents time in seconds. The responses for control and **B**-lesion simulations are similar: negative-going wave response for “correct” expected outcomes and positive-going wave to unexpected outcomes. There is a slight dip in positive response for the **B**-lesion simulation to unexpected outcomes, relative to the control (red arrow), and no evoked response for both **A**-lesioned and **A**- and **B**-lesioned subjects. This is due to the lesion reducing the precision of the evaluation likelihood and impairing the synthetic subject’s ability to distinguish between correct and incorrect evaluation. In contrast, there is a slight positive response to the unexpected stimulus, compared to the expected for both these groups (blue arrow).

We simulated local field potential responses based upon a simple form of belief updating cast as a neuronally plausible gradient descent on variational free energy (Fig. 5). A more detailed discussion of how the underlying belief updating translates into neurophysiology can be found in (Friston, FitzGerald, et al. 2017a). The results of the simulated electrophysiological responses of a single neuronal population responding to the “correct” evaluation are shown in Figure 9. The shape of the waveform completely flips when the subject is exposed to unexpected, compared to expected, evaluation. These findings are consistent for the intrinsic lesion subject, where the intensity of impairment is limited to state transitions over time (highlighted through the changes in the responses at peristimulus time 200–400 ms ; Fig. 9*A* and *C*). However, when evaluation ability is completely impaired (associated with extrinsic lesion), the subject is unable to distinguish between expected and unexpected evaluations resulting in little to no evoked response. In future work we hope to test empirically the links between stimulated evoked responses and the particular neuronal populations in (Fig. 6).

## Concluding Comments

In this paper, we used the free energy principle (a.k.a. active inference) (Friston 2019) to specify precise, quantitative roles for redundancy and degeneracy. We have shown how redundancy and degeneracy change, singly and in concert, both during learning and after damage to the cognitive system. This was achieved by associating degeneracy with entropy—and redundancy with complexity—during active inference under a given generative model (i.e., structure) and associated belief updating (i.e., function). This characterization of degeneracy and redundancy may have practical utility: 1) It measures degeneracy and redundancy in the same (natural) units of information, and 2) the same model can predict behavioral performance (i.e., accuracy) and its electrophysiological concomitants—by appealing to planning as (active) inference, when selecting a behavioral response. Using this model, we offer a principled way to assess the functional integrity of word repetition in control and patient subjects, where both behavior and electrophysiology can be recorded simultaneously. In principle, it is possible to estimate the prior beliefs (e.g., connectivity parameters) of a subject’s generative model that best explains their responses by finding the parameters of the generative model that maximizes the likelihood of the responses. This is known as computational phenotyping (Schwartenbeck and Friston 2016; Parr, Rees, et al. 2018). Furthermore, having electrophysiological predictions means that one can associate belief updating with particular brain regions via the dynamic casual modeling of neurophysiological data (see, e.g., Adams et al. (2016); Vincent et al. (2019)). This kind of approach may illuminate the mechanics of degenerate functional architecture that have previously been regarded as tight regulators of neural network performance (Cropper et al. 2016).

The formulation, presented in this paper, enabled us to run a series of simulations, where different forms of redundancy and degeneracy were measured—using both structural learning and in silico lesions. Through our numerical analyses, we show how redundant structural parameterizations (in spurious connections) incur higher complexity cost and increasingly degenerate mappings between causes and outcomes. Removing these spurious connections decreases redundancy, while removing nonspurious connections increases redundancy. These two contrasting changes in connectivity can be associated with structure learning and with lesions, respectively. Due to the distributed nature of belief updating, multiple pathways (i.e., connectivity) participate in active inference. This is consistent with lesion-deficit studies that often require distributed disconnections across multiple pathways to produce a functional deficit.

The characterization of degeneracy and redundancy—in terms of free energy components—transcends any specific generative model. The purpose of the simulations is to illustrate the consequences of this characterization in a synthetic setting (where we know the form of the generative model). However, it is worth considering the utility of this characterization and the ways in which it could be used to pose empirical questions. This would require the use of real data, to ask whether a system’s degeneracy or redundancy changes following an intervention. For example, “does a particular form of neurorehabilitative therapy reduce redundancy during recovery from a neurological insult?”.

We modeled the in silico lesions in terms of disconnections and focused on the distinction between intrinsic (within-region) and extrinsic (between-region) connections. Through this, we hoped to demonstrate the degeneracy inherent in active inference by showing that functional deficits were greater when both extrinsic and intrinsic pathways were lesioned, as opposed to either in isolation. In future work, we hope to use more comprehensive generative models—with multiple extrinsic (between-region) pathways and greater hierarchal depth—that afford degenerate structure–function mappings in cortical hierarchies. Additionally, this work lays the foundation for formalizing the mechanisms of functional recovery after brain damage, that is, quantifying changes in degenerate architectures or perilesional activity after brain insult.

## Funding

Medical Research Council (MR/S502522/1 to N.S., MR/M023672/1 to C.J.P.); Rosetrees Trust (Award Number 173346 to T.P.); Stroke Association (TSA PDF 2017/02 to T.M.H.); Wellcome Trust (Ref: 088130/Z/09/Z to K.J.F., 203147/Z/16/Z and 205103/Z/16/Z to C.J.P.).

## Notes

The authors have no disclosures or conflict of interest.

## Software Note

The generative model in these kind of simulations changes from application to application; however, the belief updates are generic and can be implemented using standard routines (here spm_MDP_VB_X.m). These routines are available as Matlab code in the SPM academic software: http://www.fil.ion.ucl.ac.uk/spm/. The code for the simulations presented in this paper can be accessed via https://github.com/ucbtns/degeneracy.

## Supplementary Material

appendix_bhaa148
