# Supplementary material for: Degeneracy and Redundancy in Active Inference
Source: Cereb Cortex. 2020 Jun 3;30(11):5750–66. doi: 10.1093/cercor/bhaa148 (PMC7899066; doi:10.1093/cercor/bhaa148)
Supplement: appendix_bhaa148 [file appendix_bhaa148.docx]

# Appendix

**A1. Model parameterisation**

Active inference rests on the tuple $\left(O,\text{ }P,\text{ }Q,\text{ }R,\text{ }S,\text{ }T,\text{ U }\right)$:

- A finite set of outcomes, $O$
- A finite set of control states or actions, $U$
- A finite set of hidden (unobservable) states, $S$
- A finite set of time-sensitive policies, $T$
- A generative process $R(o,s,u)$ that generates probabilistic outcomes $o\in O$from (hidden) states $s\in S$and action $u\in U$
- A generative model $P(o,s,\pi,\eta)=P(\pi|\eta)P(\eta)\prod_{t=1}^{T} P(o_{t}|s_{t},\eta)P(s_{t}|s_{t-1},\pi,\eta)$with parameters $\eta$, over outcomes, states, and policies $\pi\in T$, where $\pi\in\left\{0,...,\text{ }K\right\}$ returns a sequence of actions $u_{t}\text{ }=\text{ }\pi\left(t\right)$
- An approximate posterior $Q(x)=\text{ }Q\left(s_{0}|\pi\right)...\text{ }Q\left(s_{T}|\pi\right)Q\left(\pi\right)Q\left(\eta\right)$ over states, policies and parameters with expectations $\left(s_{0}^{\pi}\text{ },...,\text{ }s_{T}^{\pi},\pi,\eta\right)$

The generative process describes transitions between states in the world that generate observed outcomes. Their transitions depend on action, which depends on posterior beliefs about the next state. Subsequently, these beliefs are formed using a generative model of how observations are generated.

The generative model (based on a partially observable MDP) describes what the agent believes about the world, where beliefs about hidden states and policies are encoded by expectations. Here actions are part of the generative process in the world and policies are part of the generative model of the agent:

$$\begin{matrix} P(o_{t}|s{}_{t},A)=Cat(A) \\ P(s{}_{t}|s{}_{t-1},\pi,B)=Cat(B) \\ P(s{}_{1}|s{}_{0})=Cat(D) \\ P(\pi|\gamma)=\sigma(E-\gamma.G) \\ P(A)=Dir(a) \\ P(B)=Dir(b) \end{matrix}$$

Here, we have divided the parameters into *A*, *B*, and *γ*. In active inference, all the heavy lifting is done by minimising the free energy with respect to expectations about hidden states, policies, and model parameters. Variational free energy can be expressed as a function of the approximate posterior $Q(.)$:

$$\begin{matrix} Q(x)=\mathrm{argmin}_{Q(x)}F \\ \approx P(x|o) \end{matrix}$$

Using rules of conditional independence and variational inference, we get the following form:

$$\begin{matrix} F=E_{Q}[\ln Q(x)-\ln P(x,o)] \\ =E_{Q}[\ln Q(x)-\ln P(o)-\ln P(x|o)] \\ =E_{Q}[\ln Q(x)-\ln P(o|x)-\ln P(x)] \\ \\ =D_{KL}[Q(x)||P(x)]-E_{Q}[\ln P(o|x)] \end{matrix}$$

By taking an additional expectation over $P(o_{t}|s_{t})$we can predict future outcomes given hidden states (expected free energy):

$$\begin{matrix} G(\pi)=\sum_{t} G(\pi,t) \\ G(\pi,t)=E_{P(o_{t}|s_{t})Q(s_{t}|\pi)}[\ln Q(s_{t}|\pi)-\ln P(s_{t},o_{t})] \end{matrix}$$

**A2. Belief update equations:**

Variational updates are a self-consistent set of equalities that minimise variational free energy. We optimise expectations about hidden states (including policies and precision) through inference and optimise model parameters (likelihood, transition states). This optimisation requires finding sufficient statistics of posterior beliefs that minimise variational free energy. Under the active inference scheme, this entails calculating the solution by using a gradient descent on free energy (under some policy):

$$\begin{matrix} \varepsilon_{\tau}^{\pi}=\ln A\cdot o_{\tau}+\ln B_{\tau-1}^{\pi}s_{\tau-1}^{\pi}+\ln B_{\tau}^{\pi}s_{\tau+1}^{\pi}-\ln s_{\tau}^{\pi} \\ \varepsilon^{\gamma}=\left(\beta-\beta_{\iota}\right)+\left(\pi-\pi_{0}\right)G \end{matrix}$$

where$\beta_{\iota}=\beta+\left(\pi\text{ }-\text{ }\pi_{0}\right)G$;$\beta=1/\gamma$encodes posterior beliefs about precision;$\pi$represents the policies specifying action sequences and$\pi_{0}\text{ }=\text{ }\sigma\left(-\gamma\text{ }\cdot\text{ }G\right)$. Each *s* represents the sufficient statistics of the posterior beliefs about states at a given time (subscript) under a given policy (superscript).

By minimising these state and precision prediction errors (free energy gradients); i.e., $\varepsilon_{\tau}^{\pi}=-\partial_{s}F\text{ }$and $\varepsilon^{\gamma}\text{ }=\text{ }\partial_{\gamma}F$, we recover the posterior expectations that minimise free energy to provide Bayesian estimates of hidden variables.
